# A scanning cavity microscope

**DOI:** 10.1038/ncomms8249

**Published:** 2015-06-24

**Authors:** Matthias Mader, Jakob Reichel, Theodor W. Hänsch, David Hunger

**Affiliations:** 1 Ludwig-Maximilians-Universität München, Fakultät für Physik, Schellingstraße 4, 80799 München, Germany; 2 Max-Planck-Institut für Quantenoptik, Hans-Kopfermann-Straße 1, 85748 Garching, Germany; 3 Laboratoire Kastler Brossel, ENS/UPMC-Paris 6/CNRS, 24 rue Lhomond, F-75005 Paris, France

## Abstract

Imaging the optical properties of individual nanosystems beyond fluorescence can provide a wealth of information. However, the minute signals for absorption and dispersion are challenging to observe, and only specialized techniques requiring sophisticated noise rejection are available. Here we use signal enhancement in a high-finesse scanning optical microcavity to demonstrate ultra-sensitive imaging. Harnessing multiple interactions of probe light with a sample within an optical resonator, we achieve a 1,700-fold signal enhancement compared with diffraction-limited microscopy. We demonstrate quantitative imaging of the extinction cross-section of gold nanoparticles with a sensitivity less than 1 nm^2^; we show a method to improve the spatial resolution potentially below the diffraction limit by using higher order cavity modes, and we present measurements of the birefringence and extinction contrast of gold nanorods. The demonstrated simultaneous enhancement of absorptive and dispersive signals promises intriguing potential for optical studies of nanomaterials, molecules and biological nanosystems.

## Abstract (web summary)

Fluorescence from nanoparticles enables high-resolution optical imaging, but this approach is limited to those structures that emit light. Here, the authors demonstrate a microscope that uses a cavity to enhance the measurement of the alternative optical properties of absorption and dispersion.

## Graphical abstract

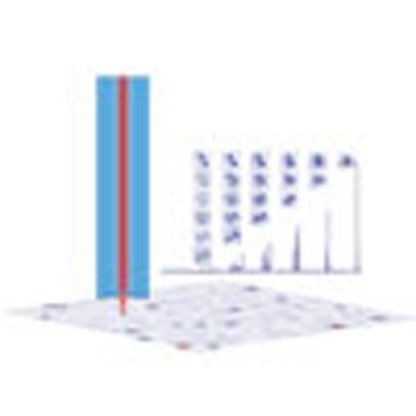

Nanoscience strives for tools that enable the characterization and imaging of nanoscale objects. Heterogeneity in particle shape, molecular morphology and microenvironment tends to wash out intrinsic properties and calls for single-particle sensitive techniques. The most common method, fluorescence microscopy, provides specific contrast and strong signals, but is limited to fluorophores that suffer from photobleaching, and does not provide information about the intrinsic optical properties of non-fluorescent samples. Detecting single-particle signals beyond fluorescence is a true challenge for small objects, since the polarizability and the absorption cross-section scale as *a*^3^ with system size *a*. To achieve the required sensitivity, the imaging techniques demonstrated to date are carefully optimized to measure one single quantity by reducing measurement noise, implementing noise rejection techniques, and by signal averaging^1^. This has enabled the imaging of weak sample absorption, for example, by photothermal microscopy^2^,^3^ or direct absorption spectroscopy^4^,^5^, as well as imaging of dispersive objects by interferometric scattering^6^,^7^. In a complementary approach, ultra-sensitive measurements can be realized by using signal enhancement within an optical cavity. Experiments such as dispersive sensing with microresonators^8^,^9^,^10^,^11^, photothermal frequency shift spectroscopy^12^ and cavity ringdown spectroscopy^13^,^14^ harness multiple round trips of light inside a cavity to enhance sensitivity. However, the experiments to date lack control over the relative position between the sample and the cavity mode, which, for most approaches, makes them incompatible with imaging and hinders quantitative analysis on a single-particle level.

In this work, we report on a versatile approach that combines cavity enhancement with high-resolution imaging and provides high sensitivity for both sample absorption and dispersion simultaneously. It is based on an open-access optical microcavity^15^,^16^,^17^,^18^,^19^ made of two highly reflective mirrors, which permits imaging a sample by raster-scanning it through a microscopic cavity mode. Fig. 1a shows the basic set-up used in our experiments. Multiple round trips of light between the mirrors accumulate loss and dispersive phase shifts caused by the sample. For a sample localized in the field maximum, the enhancement compared with a single pass amounts to 
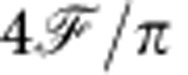
, given by the number of reflections 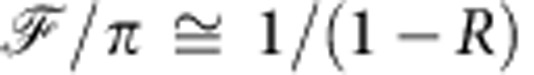 and the increased intensity owing to the standing wave in the cavity. Here 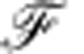 is the cavity finesse and *R* the reflectivity of both mirrors. Notably, the same enhancement is available for absorption, scattering, fluorescence and dispersive signals^20^,^21^. At the same time, the overall signal scales inversely with the mode cross-section 
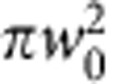
, such that a cavity that maximizes the figure of merit 
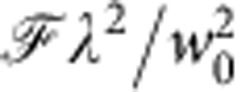
 is desired.

In the following, we report three sets of measurements that demonstrate the potential of cavity enhancement for ultra-sensitive imaging: first, we image gold nanoparticles with superior sensitivity and quantitatively evaluate their extinction cross-sections. Second, we introduce a method to improve spatial resolution by combining higher-order cavity modes and show how this can be used to surpass the diffraction limit. Finally, we make use of the simultaneous enhancement of absorptive and dispersive signals and demonstrate measurements of the polarization dependent extinction and polarizability of gold nanorods, providing a way for the detailled characterization of the polarizability tensor of a nanoscale sample.

## Results

### Scanning cavity set-up

In our approach, we use a high-finesse Fabry-Perot microcavity based on a mirror fabricated on the laser-machined endfacet of an optical fibre^15^,^22^ (see Supplementary Fig. 1). Combined with a scannable planar mirror serving as a sample holder, a cavity is formed that reaches a finesse of 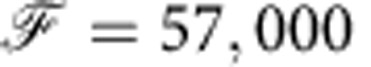 and a mode waist of *w*_0_=2.4 μm for small mirror separations.

We employ a grating-stabilized diode laser at a fixed wavelength *λ*=780 nm to probe the cavity by tuning the mirror separation across a fraction of a free spectral range of the cavity with a high-precision closed-loop nanopositioner. We detect the cavity transmission and observe several cavity modes becoming resonant at particular mirror separations. For each resonance, we evaluate the transmission, the linewidth and its position. We use an electro-optic modulator to imprint sidebands on the laser as reference markers to correct for non-linearities and mechanical noise (see Supplementary Fig. 2).

**Figure 1:**
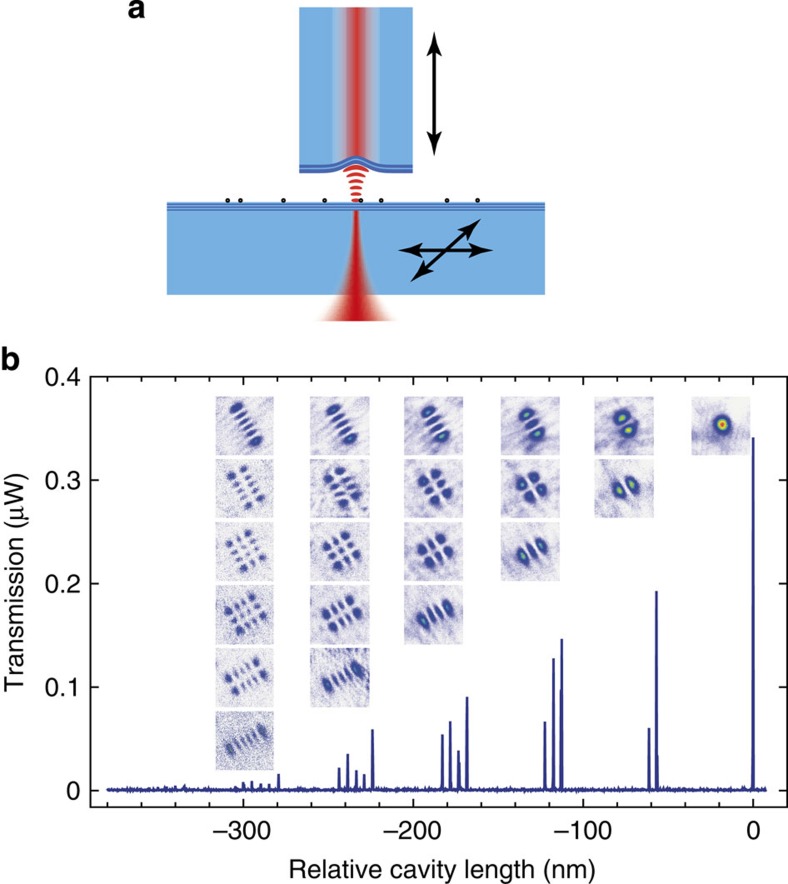
Schematic of a scanning cavity microscope. (**a**) A cavity built from a laser-machined and mirror-coated optical fibre and a planar mirror serving as a sample holder. Transverse scanning of the sample mirror is used for spatial imaging, axial scanning of the fibre for resonance tuning. (**b**) Cavity transmission signal when tuning the cavity length. The PSFs of different transverse modes (insets) are measured by scanning the cavity across a single Au nanoparticle and evaluating the resonant transmission for each mode.

Figure 1b shows a typical detector raw transmission signal when the cavity length is scanned across a few hundred nanometres. The signal contains information about additional intracavity loss, which decreases the transmission and increases the linewidth, as well as about sample polarizability, which shifts the resonance position due to the associated effective refractive index change. We raster-scan the sample mirror and record the transmission spectrum at each pixel, such that from a single measurement we can extract spatial images reflecting sample extinction and polarizability.

### Extinction cross-section of gold nanoparticles

As a first step, we demonstrate the quantitative imaging of particle extinction with high sensitivity. We study 40-nm gold nanoparticles as a reference system whose extinction cross-section can be calculated. We choose a wavelength far away from the plasmon resonance, where the extinction cross-section has dropped by nearly two orders of magnitude and amounts to ∼2% of the geometrical particle cross-section.

**Figure 2:**
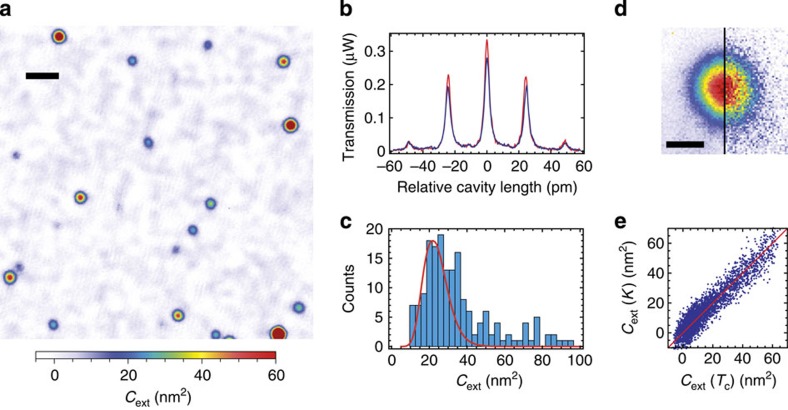
Extinction cross-section of gold nanoparticles. (**a**) Spatially resolved map of the extinction cross-section for a mirror carrying 40 nm gold nanospheres. Scale bar, 10 μm. (**b**)Transmission signal of the fundamental cavity mode with sidebands when centered on an individual nanosphere (blue) and on a clean mirror spot (red). (**c**) Histogram of the measured particle extinction cross-section (blue) and calculated distribution (red solid line). (**d**) Extinction measurement of a nanoparticle by transmission (left half) and linewidth (right half). Scale bar, 1 μm. (**e**) Pixel-by-pixel comparison between the extinction cross-section as measured by cavity transmission and linewidth.

Figure 2a shows an example for a measurement where we evaluate the resonant cavity transmission of the fundamental mode. On resonance, the cavity transmission is given by 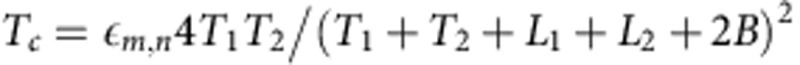. Here *T*_*i*_, *L*_*i*_, *i*={1, 2} is the respective mirror transmission and loss, which can be inferred from measurements on a clean mirror with high precision (∼5% uncertainty), and *B* is the additional loss introduced by the sample. The mode matching 
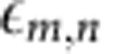
 between the fibre mode and the respective cavity mode with mode index (*m*,*n*) can be tuned by angular alignment of the fibre with respect to the plane mirror to achieve controlled coupling to modes up to order *m*+*n*∼8. From the additional loss, we can quantitatively extract the extinction cross-section of the sample, 
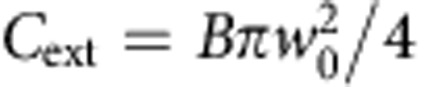
. The mode waist of the cavity can be obtained from the point spread function (PSF) observed for a point-like particle, see Fig. 2d.

The spatial map shown in Fig. 2a shows a large spread in the extinction cross-section even for a monodisperse sample. To characterize this in detail, we determine the peak extinction cross-section and the spot size of each loss feature and histogram the peak values for those features, whose size agrees with the size of the PSF of the cavity. This ensures that we mostly select only individual nanoparticles for the evaluation. We obtain a distribution peaking at *C*_ext_=25 nm^2^ with a full width at half maximum of 22 nm^2^. We compare the measurement to a calculation of the extinction cross-section, where we take into account absorption and scattering^23^, the effect of the mirror surface^24^ and surface-scattering induced damping^25^ (see Supplementary Note 1). For 41-nm gold particles on a fused silica surface at a wavelength of 780 nm, we calculate *C*_ext_=22 nm^2^. Together with the specified size distribution, we obtain a calculated distribution that reproduces the data very well without any free parameter, see Fig. 2c. This underlines the potential of our technique for quantitative sample characterization.

In the same manner, we can also evaluate the resonance linewidth *κ*=*c*(*T*_1_+*T*_2_+*L*_1_+*L*_2_+2*B*)/(4*d*), with *c* the speed of light and *d* the length of the cavity including penetration of the field into the dielectric mirror. While the linewidth is immune to intensity fluctuations and thereby shows less drifts, the shot-to-shot variation is larger due to mechanical noise coupling to the cavity. When comparing the extinction extracted from linewidth and transmission data, we observe the expected linear correlation with unity slope, see Fig. 2e.

We emphasize that, due to the cavity, the small extinction cross-section of 25 nm^2^ leads to a large (17%) change in the raw transmission signal as shown in Fig. 2b. This is in contrast to the expected single-pass signal of 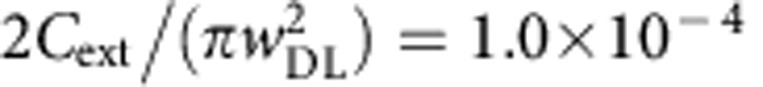 for a diffraction-limited microscope, where *w*_DL_≈*λ*/2. In comparison, the cavity enhances the signal by a factor of 1,700, in agreement with the expected factor of 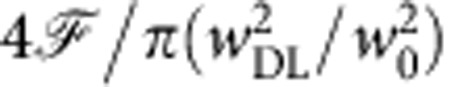. In the measurements shown, we achieve a sensitivity for extinction cross-sections of 0.5 nm^2^, limited by spatial variation of the background on a clean mirror (see Supplementary Fig. 3). The achieved sensitivity corresponds to the extinction cross-section of 2 nm gold spheres at the plasmon resonance and is comparable to, for example, typical values for cross-sections of single-quantum emitters at room temperature^5^.

### Resolution improvement with higher-order transverse modes

As a next step, we demonstrate a method to improve the spatial resolution by using higher-order cavity modes^26^,^27^,^28^. Higher transverse modes carry larger transverse momentum and can thus be used to resolve smaller structures, similar to the concept used in structured illumination microscopy^29^. Here we follow the principle of constructing a squeezed state of the quantum harmonic oscillator, where a suitable superposition of Hermite–Gaussian (HG) states yields squeezed states with reduced position uncertainty and correspondingly increased momentum uncertainty. We adopt this approach to optical cavity modes, and construct an effective mode with a spatial distribution that is smaller than the Gaussian fundamental mode. Since the HG modes are not simultaneously resonant due to the mode-dependent Gouy phase, only an incoherent superposition is possible, in contrast to the coherent superposition for squeezed states. Still, one can find a suitable expansion of the intensity distributions that closely approximates a squeezed state. Considering a one-dimensional situation to illustrate the principle, we use


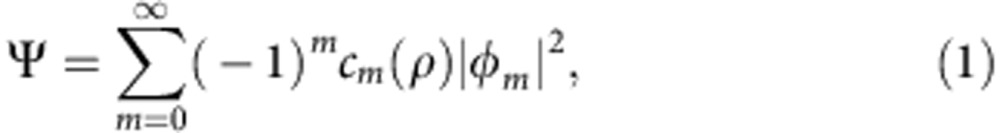


with coefficients *c*_*m*_(*ρ*)=*m*!/(2^*m*^(*m*/2)!^2^) × tanh^*m*^(*ρ*)/cosh(*ρ*) containing the ‘squeezing strength' *ρ*, and the HG modes 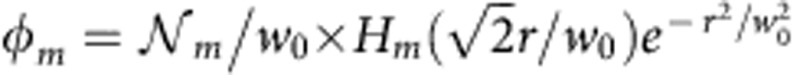 with the Hermite polynomials *H*_*m*_ and the normalization 
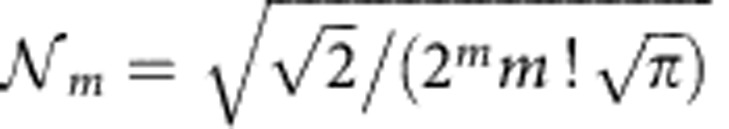
 (ref. 30). We also include odd HG modes in a way that the linear combination adds the even and subtracts the odd modes. This has almost the same effect as the interference that is present for the coherent superposition.

**Figure 3:**
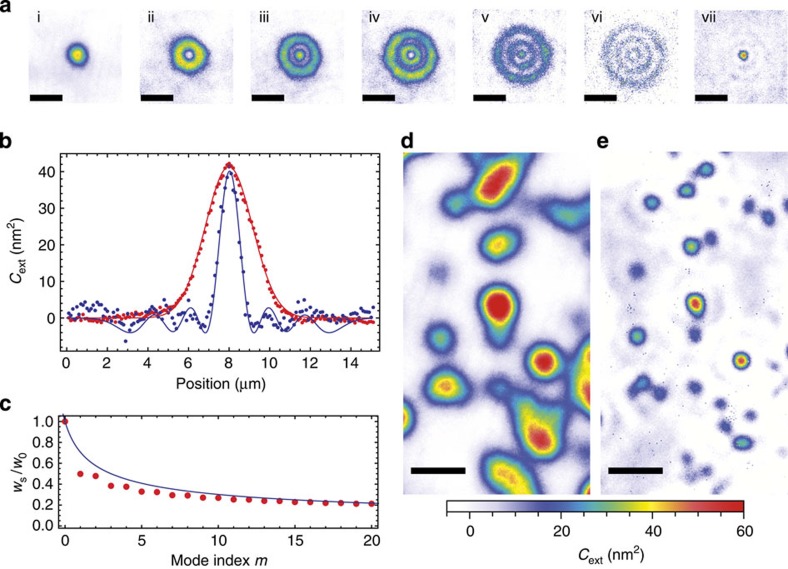
Resolution enhancement by higher transverse modes. (**a**) Extinction measurement of a single particle using the fundamental mode (i) and the first five higher transverse-mode families (ii)–(vi), combined to yield an enhanced-resolution mode (vii). Scale bars, 5 μm (**b**) Averaged section (5 rows) through the fundamental mode (red dots) and enhanced-resolution mode (blue dots) together with a fit (solid lines). (**c**) Achievable spatial resolution improvement, showing the ratio of the reduced (*w*_*s*_) and initial (*w*_0_) mode waist as a function of the maximal mode order included. (**d**) Extinction map of 40 nm Au NPs. (**e**) Enhanced-resolution map of the same area using the higher mode orders up to *m*+*n*=3. Scale bars, 10 μm

We evaluate the localization of Ψ by inferring the position *w*_*s*_ where Ψ=1/*e*^2^ for different numbers of HG modes contributing. At this stage, we remain in the paraxial approximation, and discuss deviations below. We find that the resolution improves according to 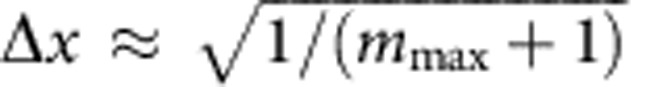, where *m*_max_ is the largest mode order included, see Fig. 3c. This is in accordance with the expected scaling that results from the increase of the number of transverse field nodes ∝ *m* and the increase of the mode radii 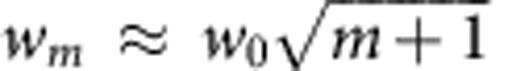. In consequence, for an optical system where the numerical aperture (NA) is not fully used, resolution can be increased at least down to the diffraction limit. This is the case for optical microcavities, which can have an NA approaching unity for small mirror separation, but where the waist of the fundamental mode remains larger than the diffraction limit because the mirror radius of curvature is large compared to the mirror separation.

Figure 3 shows the experimental realization of this concept, which involves an extension of the above principle to two dimensions. In the measurements, we record the transmission and linewidth of all different modes within a single measurement by recording traces such as the one shown in Fig. 1b for each pixel, where within a few microseconds, all modes are probed and recorded sequentially. As a first step, we evaluate the transmission of cavity modes up to the fifth order (*m*+*n*=5), comprising all 15 modes shown in Fig. 1b. We sum over all transverse modes belonging to one mode order, which leads to rotationally symmetric, concentric ring shapes, see Fig. 3a. Combining these modes according to equation (1), we arrive at the enhanced-resolution PSF shown in Fig. 3a. We set *ρ*=2, which at the same time optimizes the spatial resolution and minimizes oscillations of the outer part of the PSF. In Fig. 3b, an averaged section through |*φ*_00_|^2^ and Ψ as well as fits to the measured values using the described model are presented. The enhanced-resolution PSF has an 1/*e*^2^ radius of 0.87 μm, a factor of 2.7 smaller than the fundamental mode, and a factor of 2.2 away from the diffraction limit of *λ*/2=390 nm.

Figure 3d,e shows the improved resolution when imaging 40 nm Au nanospheres in direct comparison. Features that are not resolvable with the fundamental mode become clearly separated, and the background is only weakly modulated due to the oscillations of the outer part of the PSF. At the same time, the high sensitivity, the enhancement factor and the quantitative character of the measurement is maintained.

Owing to its relevance for imaging in general, we briefly touch on the achievable limits of resolution when considering a rigorous calculation of the vector field without relying on the paraxial approximation^31^,^32^. We calculate the focal field of HG modes when projected from the far field. We find that only the *φ*_01/10_ modes are useful for resolution enhancement, since higher modes are not suited for input aperture overfilling. A calculation of Ψ=|*φ*_00_|^2^−0.6(|*φ*_01_|^2^+|*φ*_10_|^2^) yields an effective PSF with a first zero crossing at Δ*x*≈0.39*λ*/NA, such that the resolution can be improved by a factor 1.5 below the diffraction limit Δ*x*≈0.61*λ*/NA. The scheme could be easily implemented in standard confocal microscopes by using two illumination paths, providing both standard illumination for a (near) Gaussian spot, and illumination with an azimuthally polarized doughnut mode. Acquisition of two images and subsequent subtraction provides superresolution with little overhead also for non-fluorescent objects.

### Extinction contrast and birefringence of Au nanorods

More detailed information about the optical properties can be obtained when studying both absorption and dispersion of a sample. Furthermore, many samples of interest lack spherical symmetry, and their optical response depends on the relative orientation between the laser polarization and the eigenaxes of the samples' polarizability tensor. The resulting angle-dependent extinction and dispersion give rise to observable extinction contrast and birefringence. However, for nanoscale samples, the signals are minute and their simultaneous imaging has been out of reach so far. Here we show that by monitoring resonance frequency shifts of the cavity, we can image dispersive properties of a sample in parallel with sample extinction in a polarization-sensitive way.

As an example, we study gold nanorods of size 34 × 25 × 25 nm^3^, which are expected to show extinction contrast and birefringence due to their anisotropic shape. Given the cylindrical symmetry, the complex polarizability tensor simplifies to the components parallel and perpendicular to the long axis, 
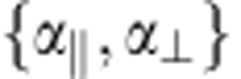
. We demonstrate simultaneous imaging of the extinction contrast and birefringence by measuring the linewidths and frequency splitting of two orthogonally polarized cavity modes. This provides information about the orientation and shape and thus the internal structure of the sample.

**Figure 4:**
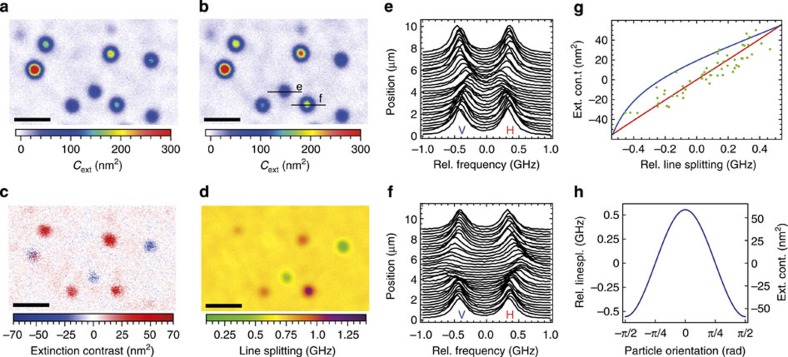
Extinction contrast and birefringence imaging. Extinction map of Au-nanorods for *H*—polarized (**a**) and *V*—polarized (**b**) light. (**c**) Extinction contrast 
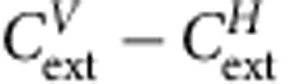
. (**d**) Absolute splitting of the two orthogonally polarized fundamental cavity modes. Scale bars, 10 μm. (**e**,**f**) Transmission signal of the cavity while scanning over the nanorods denoted in **b**. (**g**) Correlation between relative line splitting and extinction contrast. Measured values (green dots), calculated correlation assuming a fixed (red solid line) and variable (blue solid line) cavity mode orientation are shown. (**h**) Relative line splitting and extinction contrast in dependence of the orientation of the nanorod.

In our experiment, we have to take into account the intrinsic mode splitting present in our cavity. Owing to ellipticity of the laser-machined mirror surface profiles, the modes of the cavity are split into a linear polarization doublet (denoted by *H→*fast, *V→*slow), whose axis and splitting is determined by the mirror shape^33^. By setting the input polarization, we can excite both modes as shown in Fig. 4e,f, and evaluate their response to study polarization effects.

Figure 4a,b shows the extinction cross-section for the two polarization modes as inferred from the cavity linewidth. We evaluate the difference of the extinction cross-section 
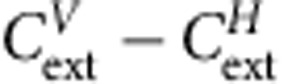
 and find significant values for most of the particles, see Fig. 4c. Fig. 4h displays the expected values for 
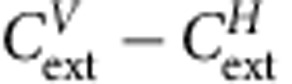
 as a function of the particle orientation, showing good agreement with the observed range (see Supplementary Note 1).

From the same measurement, we infer the birefringence of the sample by monitoring the separation of the polarization modes of the cavity. The differential frequency shift Δ*ω* of the cavity resonance doublet due to a birefringent particle is found to be^34^,^35^


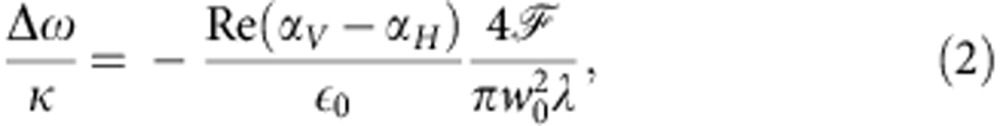


where we have normalized to the bare cavity linewidth *κ* and used the projection of the polarizability eigenaxes onto the cavity eigenaxes. For a nanorod with the long axis in the plane of the mirror, this is given by 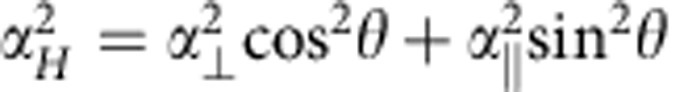, 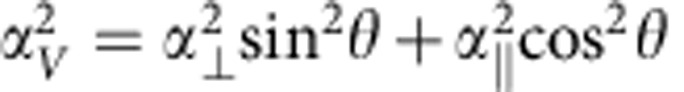, with the angle *θ* between the orientation of the long particle axis and the *V* cavity mode.

Figure 4d shows a spatial map of the measured birefringence signal with the constant offset of 740 MHz originating from the intrinsic cavity birefringence. Figure 4h displays a calculation of the relative cavity mode splitting in presence of a gold nanorod as a function of particle orientation, where we assume a polarizability volume 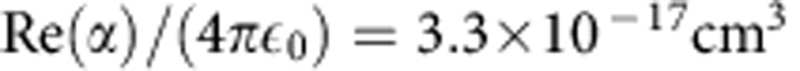 (2.1 × 10^−17^cm^3^) for the long (short) axis (see Supplementary Note 1).

Figure 4e,f shows two example signatures of nanorods that are mostly orthogonal (parallel) to the slow cavity eigenaxis, and thereby reduce (increase) the intrinsic birefringence. At the same time, the birefringence is correlated with the extinction difference, where the mode parallel to the long particle axis is affected more strongly.

In Fig. 4g, we show the evaluated extinction difference and the relative dispersive frequency shift of a large number of nanorods. We observe good agreement between the expected and measured range of values. The correlation of the two quantities shows a linear relation and agrees with the prediction, which is again calculated without free parameters. With 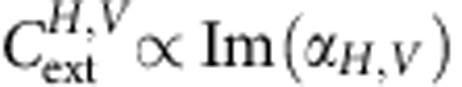 and Δ*ω*∝Re(*α*_*H*,*V*_), the correlation provides a detailed characterization of the polarizability tensor of the sample. Notably, the data yield good agreement with the expected signal when assuming a fixed orientation of the cavity eigenaxis. This is in contrast to the expectation that the cavity eigenmodes are rotated by the presence of sample birefringence when *θ*≠0 (refs 36, 37). The absence of rotation is furthermore confirmed by evaluating the cavity transmission after a polarizing beam splitter (see Supplementary Fig. 4). This suggests that the geometry-induced mode splitting fixes the eigenmode axes.

In the measurements on line splitting, we achieve a noise floor of 9-MHz rms, yielding a sensitivity for a polarizability volume difference of 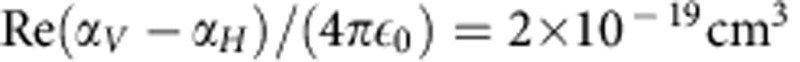. Since we probe the nanorods far away from the plasmon resonance, their polarizability is comparable to the value of dielectric objects of same size. The demonstrated sensitivity should thus allow spatial imaging of, for example, individual macromolecules with a size down to a few tens of nanometres.

## Discussion

We have demonstrated a versatile technique for sensitive optical imaging based on an open-access, scannable microresonator. The combination of high spatial resolution, simultaneous high sensitivity for absorption and birefringence and the quantitative nature of the signals promises great potential for label-free biosensing, characterization of nanomaterials, particle sizing and spectroscopy of quantum emitters on a single-particle level.

In addition, our method could provide new insight into the microscopic properties of low loss mirrors, as used, for example, for gravitational wave detectors, cavity QED experiments and laser gyroscopes. Finally, fluorescence nanoscopy methods such as stimulated emission depletion microscopy^38^ could be implemented, where the near-ideal shape of the higher order modes, the intrinsic power enhancement, and Purcell enhancement of spontaneous emission could add significant benefit.

Our method is still open for substantial improvements of the sensitivity. The current limitation due to spatial background variation could be overcome by differential measurements, for example, before and after application of the sample. Furthermore, the signal enhancement can be further increased by improved cavities, where we expect mode waists *w*_0_<*λ* and a finesse 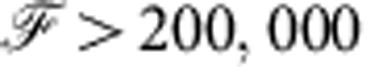 to be achievable. In addition, our method could be combined with noise reduction techniques, such that the relative noise level of 2 × 10^−2^ reached in most of the measurements shown here, could be reduced potentially down to 10^−6^ (refs 5, 14).

## Methods

### Experimental set-up

The microcavity is based on a micromirror on the endfacet of an optical fiber, which has a laser-machined concave depression with an effective radius of curvature of 60 μm. It is coated with a dielectric mirror with *R*=99.9976%. A planar 1/2" mirror with *R*=99.9914% serves as a sample holder. The cavity is typically operated at a length of *d*≈30*λ*/2 to avoid transverse-mode coupling^39^,^40^, where it has a linewidth of 245 MHz and corresponding quality factor *Q*=1.57 × 10^6^. The cavity is probed with an external cavity diode laser at a wavelength of 780 nm with a linewidth below 1MHz (Toptica DL pro), coupled to the cavity fiber. The laser is phase-modulated with an EOM (Newfocus 4221) to generate sidebands used as frequency markers for linewidth and line splitting measurements. We excite the cavity such that the power circulating inside the cavity remains smaller than 10 mW. The transmitted light is split up by a polarizing beam splitter aligned along the polarization axes of the resonator. The light is detected with two photodiodes (Thorlabs APD120) and recorded with an oscilloscope (LeCroy HRO66Zi). The laser intensity can be adjusted for each cavity mode order with an AOM. The length of the cavity is scanned with a closed-loop piezo-linear actuator (Physikinstrumente LISA P-753, E-712). Its absolute length is determined from white light transmission spectra, where we use a superluminescent diode (EXALOS EXS7505–8411) and analyze the transmitted light with a spectrometer (Ocean Optics HR4000). The length is stabilized by comparing the resonance positions of the 780 nm probe laser and a homebuilt external cavity diode laser at a wavelength of 969 nm, where the cavity has a finesse below 1. Both lasers are intensity but not frequency stabilized.

The 1/2" plane mirror is scanned transversally with an *xy* piezo scanner (Physikinstrumente P-734, E-712). A sketch of the optics can be seen in Supplementary Fig. 1.

### Data analysis

The recorded cavity resonances are fitted online with a sum of Lorentzians. The linewidth and the line splitting is determined by using sidebands modulated on the laser as frequency markers. The measurement time amounts to ∼100 ms per pixel for the data shown, including the time needed for positioning, the length scan, data transfer and data evaluation. In principle, the time response of the cavity is the fundamental limiting factor, with a typical time constant of 10 ns. We expect that with a cavity locked on resonance while scanning transversally, this limit can be approached.

The transmission data are normalized such that the most probable value for an empty mirror is 1. The normalized data are then rescaled with the calculated transmission of an empty cavity. For evaluating the amplitudes of the extinction cross-section or line shift of spatial images, we fit a Gaussian to each circular object. For further analysis, we select only particles whose image size corresponds to the PSF, to exclude larger particles or clusters of particles.

### Nanoparticles and sample preparation

We spincast 100 μl of a 1:33 dilution of colloidal gold nanospheres with a diameter of 40 nm (British Biocell International, plasmon resonance at 530 nm) or colloidal nanorods with a size of 25 × 25 × 34 nm^3^ (Strem Chemicals, plasmon resonance at 550 nm) onto a clean cavity mirror. Supplementary Fig. 5 shows a scanning electron microscope image of a sample of gold nanospheres.

## Additional information

**How to cite this article**: Mader, M. *et al.* A scanning cavity microscope. *Nat. Commun.* 6:7249 doi: 10.1038/ncomms8249 (2015).

## Supplementary Material

Supplementary Information Supplementary Figures 1-5, Supplementary Note 1 and Supplementary References

## References

[b1] Yurt A., Daaboul G. G., Connor J. H., Goldberg B. B. & Ünlü M. S. Single nanoparticle detectors for biological applications. Nanoscale 4, 715–726 (2012). 22214976 10.1039/c2nr11562j PMC3759154

[b2] Boyer D., Tamarat P., Maali A., Lounis B. & Orrit M. Photothermal imaging of nanometer-sized metal particles among scatterers. Science 297, 1160–1163 (2002). 12183624 10.1126/science.1073765

[b3] Cognet L. *et al.* Single metallic nanoparticle imaging for protein detection in cells. Proc. Natl Acad. Sci. USA 100, 11350–11355 (2003). 13679586 10.1073/pnas.1534635100 PMC208760

[b4] Arbouet A. *et al.* Direct measurement of the single-metal-cluster optical absorption. Phys. Rev. Lett. 93, 127401 (2004). 15447305 10.1103/PhysRevLett.93.127401

[b5] Celebrano M., Kukura P., Renn A. & Sandoghdar V. Single-molecule imaging by optical absorption. Nat. Photonics 5, 166–169 (2011).

[b6] Ortega-Arroyo J. & Kukura P. Interferometric scattering microscopy (iSCAT): New frontiers in ultrafast and ultrasensitive optical microscopy. Phys. Chem. Chem. Phys. 14, 15609–15928 (2012). 22996289 10.1039/c2cp41013c

[b7] Piliarik M. & Sandoghdar V. Direct optical sensing of single unlabeled small proteins and super-resolution microscopy of their binding sites. Nat. Commun. 5, 4495 (2014). 25072241 10.1038/ncomms5495

[b8] Vollmer F. & Arnold S. Whispering-gallery-mode biosensing: label-free detection down to single molecules. Nat. Methods 7, 591–596 (2008). 18587317 10.1038/nmeth.1221

[b9] Zhu J. *et al.* On-chip single nanoparicle detection and sizing by mode splitting in an ultrahigh-Q microresonator. Nat. Photonics 4, 46–49 (2009).

[b10] Vollmer F. & Yang L. Label-free detection with high-Q microcavities: a review of biosensing mechanisms for integrated devices. Nanophotonics 1, 267–291 (2012). 10.1515/nanoph-2012-0021 PMC4764104 26918228

[b11] Baaske M. D., Foreman M. R. & Vollmer F. Single-molecule nucleic acid interactions monitored on a label-free microcavity biosensor platform. Nat. Nanotechnol. 9, 933–939 (2014). 25173831 10.1038/nnano.2014.180

[b12] Heylman K. D., Knapper K. A. & Goldsmith R. H. Photothermal microscopy of nonluminescent single particles enabled by optical microresonators. J. Phys. Chem. Lett. 5, 1917–1923 (2014). 10.1021/jz500781g 26273873

[b13] Berden G., Peeters R. & Mejer G. Cavity ring-down spectroscopy: experimental schemes and applications. Int. Rev. Phys. Chem. 19, 565–607 (2000).

[b14] Ye J., Ma L.-S. & Hall J. L. Ultrasensitive detections in atomic and molecular physics: demonstration in overtone spectroscopy. J. Opt. Soc. Am. B 15, 6–15 (1998).

[b15] Hunger D. *et al.* Fiber Fabry-Perot cavity with high finesse. New J. Phys. 12, 065038 (2010).

[b16] Toninelli C. *et al.* A scanning microcavity for in situ control of single-molecule emission. Appl. Phys. Lett. 97, 021107 (2010).

[b17] Muller A., Flagg E. B., Lawall J. R. & Solomon G. S. Ultrahigh-finesse, low-mode-volume Fabry-Perot microcavity. Opt. Lett. 35, 2293–2295 (2010). 20596224 10.1364/OL.35.002293

[b18] Greuter L. *et al.* A small volume tunable microcavity: Development and characterization. Appl. Phys. Lett. 105, 121105 (2014).

[b19] Dolan P. R., Hughes G. M., Grazioso F., Patton B. R. & Smith J. M. Femtoliter tunable optical cavity arrays. Opt. Lett. 35, 3556–3558 (2010). 21042348 10.1364/OL.35.003556

[b20] Motsch M., Zeppenfeld M., Pinkse P. W. H. & Rempe G. Cavity-enhanced rayleigh scattering. New J. Phys. 12, 063022 (2010).

[b21] Tanji-Suzuki H. *et al.* Interaction between atomic ensembles and optical resonators: classical description. Preprint at http://arXiv.org/abs/1104.3594 (2011).

[b22] Hunger D., Deutsch C., Barbour R. J., Warburton R. J. & Reichel J. Laser micro-fabrication of concave, low-roughness features in silica. AIP Adv. 2, 012119 (2012).

[b23] van de Hulst H. Light Scattering by Small Particles Dover Books on Physics Series Dover Publications (1957).

[b24] Wind M. M., Vlieger J. & Bedeaux D. The polarizability of a truncated sphere on a substrate i. Phys. A 141, 33–57 (1987).

[b25] Muskens O. L., Billaud P., Broyer M., Del Fatti N. & Vallée F. Optical extinction spectrum of a single metal nanoparticle: quantitative characterization of a particle and of its local environment. Phys. Rev. B 78, 205410 (2008).

[b26] Horak P. *et al.* Optical kaleidoscope using a single atom. Phys. Rev. Lett. 88, 043601 (2002). 11801120 10.1103/PhysRevLett.88.043601

[b27] Dorn R., Quabis S. & Leuchs G. Sharper focus for a radially polarized light beam. Phys. Rev. Lett. 91, 233901 (2003). 14683185 10.1103/PhysRevLett.91.233901

[b28] Gigan S., Lopez L., Treps N., Matre A. & Fabre C. Image transmission through a stable paraxial cavity. Phys. Rev. A 72, 023804 (2005).

[b29] Gustafsson M. G. L. *et al.* Three-dimensional resolution doubling in wide-field flourescence microscopy by structured illumination. Biophys. J. 94, 4957–4970 (2008). 18326650 10.1529/biophysj.107.120345 PMC2397368

[b30] Gerry C. C. & Knight P. Introductory Quantum Optics Cambridge Univ. Press (2005).

[b31] Richards B. & Wolf E. Electromagnetic diffraction in optical systems: II. structure of the image field in an aplanatic system. Proc. Roy. Soc. A 15, 358–379 (1959).

[b32] Novotny L. & Hecht B. Principles of Nano-Optics Cambridge Univ. Press (2006).

[b33] Uphoff M., Brekenfeld M., Rempe G. & Ritter S. Frequency splitting of polarization eigenmodes in microscopic Fabry-Perot cavities. New J. Phys. 17, 013053 (2015).

[b34] Arnold S., Khoshsima M., Teraoka I., Holler S. & Vollmer F. Shift of whispering-gallery modes in microspheres by protein adsorption. Opt. Lett. 28, 272–274 (2003). 12653369 10.1364/ol.28.000272

[b35] Noto M., Keng D., Teraoka I. & Arnold S. Detection of protein orientation on the silica microsphere surface using transverse electric/transverse magnetic whispering gallery modes. Biophys. J. 92, 4466–4472 (2007). 17400701 10.1529/biophysj.106.103200 PMC1877779

[b36] Brandi F. *et al.* Measurement of the phase anisotropy of very high reflectivity interferential mirrors. Appl. Phys. B 65, 351–355 (1997).

[b37] Moriwaki S., Sakaida H., Yuzawa T. & Mio N. Measurement of the residual birefringence of interferential mirrors using Fabry-Perot cavity. Appl. Phys. B 65, 347–350 (1997).

[b38] Hell S. Far field optical nanoscopy. Science 316, 1153–1158 (2007). 17525330 10.1126/science.1137395

[b39] Klaassen T., de Jong J., van Exter M. & Woerdman J. P. Transverse mode coupling in an optical resonator. Opt. Lett. 30, 1959–1961 (2005). 16092232 10.1364/ol.30.001959

[b40] Benedikter J., Hümmer T., Mader M., Hänsch T. & Hunger D. Resonant mode mixing and diffraction loss in tunable fabry-perot microcavities. Preprint at http://arxiv.org/abs/1502.01532. (2015).

