# Supplementary material for: A scanning cavity microscope
Source: Nat Commun. 2015 Jun 24;6:7249. doi: 10.1038/ncomms8249 (PMC4491170; doi:10.1038/ncomms8249)
Supplement: Supplementary Information — Supplementary Figures 1-5, Supplementary Note 1 and Supplementary References [file ncomms8249-s1.pdf]

Supplementary Figure 1: Micro-Machined Fiber

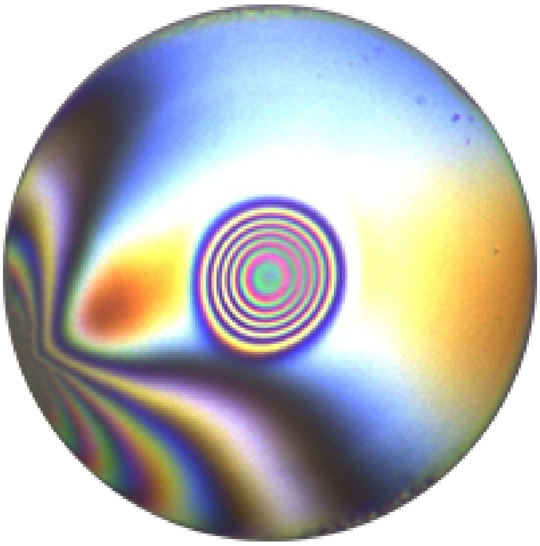

Endfacet of the laser-machined fiber used in the experiment. Picture taken with a home-buit white-light interferometric microscope. The fiber endfacet is mirror coated with a commercial IBS coating (R=99.9976%, T=L=12 ppm) from ATF, Boulder.

Supplementary Figure 2: Scanning Cavity Setup

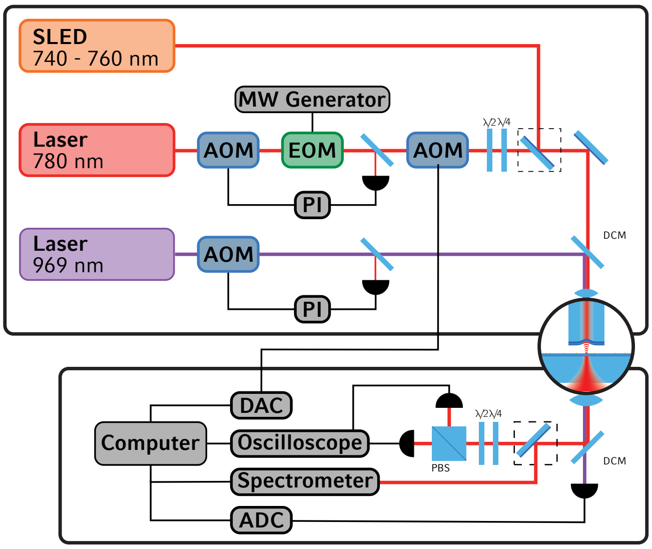

Probe laser The cavity is probed by a grating stabilized external cavity diode laser (TOPTICA DLpro) at a wavelength of 780 nm. The power of the laser ist stabilized to a relative noise level of 10^{-4} by a feedback loop consisting of an AOM, a PI controller and a photodiode.

The laser is phase-modulated with a resonant EOM (Newfocus 4421) at a frequency of 1.748 GHz to generate sidebands which are used as frequency markers for cavity linewidth measurements as well as for measurements of the linesplitting of the two orthogonally polarized cavity modes.

As different transverse modes of the cavity have significantly different mode matching to the input light, the power of the probe light can be adjusted by a second AOM. It is possible to switch the intensity in a way that all recorded resonances have nearly the same intensity on the detectors in order to fully use the dynamic range of the detector and the oscilloscope.

The transmitted light is collected by an achromatic lens. The two orthogonally polarized modes of the cavity are separated by a polarizing beamsplitter. The light is detected by two APD's (Thorlabs APD120A). The signal is then recored by an oscilloscope (LeCroy HRO66Zi).

Cavity length measurement To measure and adjust the length of the cavity precisely, the cavity can be probed with a superluminescent LED (EXALOS EXS7505-8411). By analyzing the transmitted light on a spectrometer (Ocean Optics HR4000), we can deduce the absolute length of the cavity including the field penetration into the dielectric mirrors from the spectral spacing of subsequent resonances.

Stabilizing the cavity length While scanning the surface of the plane mirror, the cavity length can change because of thermal and mechanical drifts, or a wedged plane mirror. If the cavity length changes, the relative position of the resonances

compared to the beginning of the cavity length scan changes, and thus the resonances of interest can shift out of the data recording window.

To compensate this, we use a second laser (homebuilt external cavity diode laser) at a wavelength of 969 nm. At this wavelength the finesse of the resonator has dropped below 1. Thus, the resonator is not sensitive to weak absorbers. By comparing the actual position of a cavity resonance with a target value, we can readjust the start position of the length scan and thereby stabilize the average length of the cavity to within a few nanometers over days.

Supplementary Figure 3: Plane Mirror

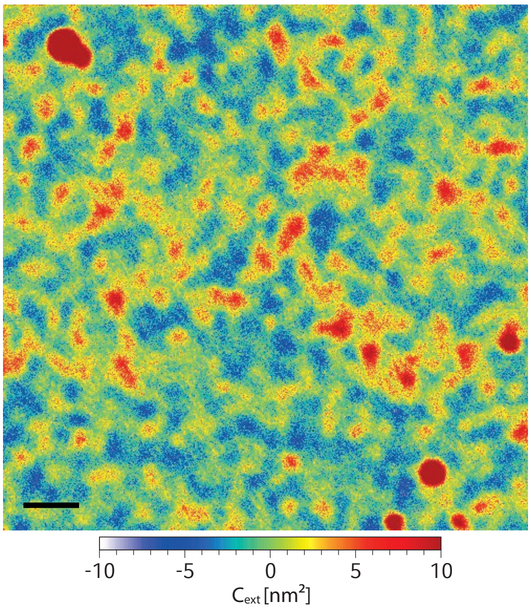

The plane mirrors used as sample holders in our experiments are superpolished fused silica substrates, coated with a high reflective dielectric coating (R=99.9914%, T=60 ppm, L=26 ppm) with SiO_2 as terminating layer, while the back side is anti-reflection coated. The coatings were manufactured by LAYERTEC, Mellingen.

The figure shows a map of extinction cross sections measured for a clean plane mirror. The typical variation of the mirror loss amounts to 0.2 ppm rms, corresponding to an extinction cross section of $0.5\,\mathrm{nm}^2$ for the cavity parameters used in the experiments.

Supplementary Figure 4: Birefringence of a Cavity

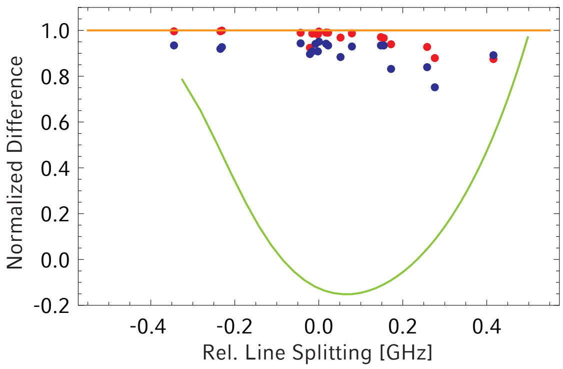

We observe that each mode of the cavity is split up into two orthogonally polarized modes. Uphoff et al. [1] show that the small ellipticity of the micromachined mirror on the fiber endfacet leads to a differential phaseshift between light polarized along the two half axes of the resonator. Thus each mode of the cavity is split up into a doublet. We exploit this for measuring anisotropy of extinction and cavity lineshift of single gold nanorods. For our theoretical considerations, we calculate the extinction cross section and the cavity line shift using a projection of the polarizability tensor of the particle onto the corresponding polarization axis.

We do not observe a rotation of the orientation of the cavity eigenaxes in the presence of particle birefringence, as predicted e.g. by Moriwaki et. al. [2] or Brandi et. al. [3]. If rotation would be induced, a second resonance peak would appear at the output ports of the polarizing beamsplitter, which is aligned with respect to the unperturbed cavity modes.

In order to prove this effect, we first fit the sum of both ports to get the position and width of each peak. In a second step, we fit each signal separately with a double Lorentzian, using the positions and widths from the first step in order to get the amplitudes of the two peaks, if they would appear.

The figure shows the measured difference of the two amplitudes of peaks for the two output ports of the beamsplitter. The values are normalized to the sum of both amplitudes. The data agree quite well with a model assuming no change of the polarization inside the cavity, while they obviously deviate from a model that assumes the nanoparticle changing the polarization orientation inside the cavity.

Supplementary Figure 5: SEM Image of Gold Nano-Spheres

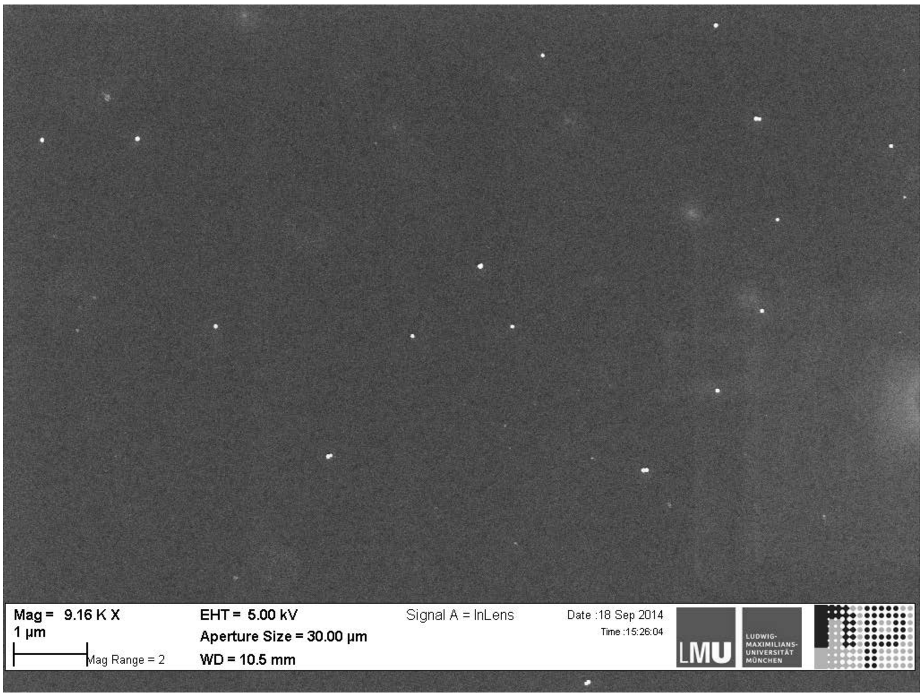

Scanning electron microscope image of mirror with gold nanospheres used in the experiments.

Supplementary Note 1: Optical Properties of a Metal Nanoparticle

For the interpretation of the experiments shown in this work, a detailed understanding of the optical properties of the metal nanoparticles is crucial. In the following, different effects that influence the properties are discussed.

The dielectric constants of the particle, its surrounding and the surfaces in the vicinity of the particle are denoted as follows: ϵ_1 denotes the dielectric constant of the surrounding environment, ϵ_2 the dielectric constant of a surface close to the particle and ϵ_3 gives the dielectric constant of the particle it self. ϵ_0 is the vacuum dielectric constant.

Polarizability of a nanoparticle

Polarizability is the key quantity to describe the optical properties of a particle. In the following, basic formulas are presented:

Spherical particles For spherically symmetric and isotropic particles, the polarizability is given by the Clausius-Mossotti law:

$$\alpha = 3\epsilon_0 V_p \frac{\epsilon_3 - \epsilon_1}{\epsilon_3 + 2\epsilon_1} \tag{1}$$

where V_p is the volume of the particle.

Ellipsoids For ellipsoidal nanoparticles, the polarizability is described by a tensor. The main values of the tensor can be calculated as follows [4]:

$$\alpha_j = \frac{V_p \epsilon_0}{L_j + \frac{1}{\epsilon_3 - 1}}, \tag{2}$$

where j denotes the axis, V_p the volume of the particle and L_j is a dimensionless parameter depending on the ratios of the semiaxes a_1, a_2 and a_3 of the particle. For arbitrary ratios of those axes, L_1 is given by

$$L_1 = \int_0^\infty \mathrm{d}s \frac{a_1\, a_2\, a_3}{2(s + a_1^2)^{3/2}\,(s + a_2^2)^{1/2}\,(s + a_3^2)^{1/2}}, \tag{3}$$

L_2 and L_3 are obtained by cyclical permutation. Furthermore, the relation

$$L_1 + L_2 + L_3 = 1 \tag{4}$$

has to be fulfilled.

For prolate ellipsoids like nanorods, where $a_1 > a_2, a_3$ and $a_2 = a_3$, the problem simplifies to

$$e^2 = 1 - \frac{a_2^2}{a_1^2}, \quad L_1 = \frac{1 - e^2}{e^2}(-1 + \frac{1}{2e} \ln \frac{1 + e}{1 - e}). \tag{5}$$

For nanorods that are not aligned along the polarization of the probe light, we rotate the polarizability tensor and project it onto the polarization axes. We do the projection for the imaginary and real part separately.

Correction of the dielectric constant because of the small size

It turns out that the dielectric constant of gold ϵ_3 has to be modified with respect to the bulk value (ϵ_{bulk}) [5] in order to describe very small particles accurately [6]:

$$\epsilon_3(\lambda) = \epsilon_{bulk}(\lambda) + \frac{\omega_p^2}{\omega(\omega + i\gamma_{bulk})} - \frac{\omega_p^2}{\omega(\omega + i\gamma)}, \tag{6}$$

with the metal plasma frequency ω_p and the electron scattering rates in the bulk (γ_{bulk}) and confinded metal (γ), where

$$\gamma = \gamma_{bulk} + 2gv_F/D \tag{7}$$

with v_F the Fermi velocity, D the particle diameter and g a proportionality factor in the order of 1.

At a wavelength of 780 nm and $g = 1.4$ we find an extinction cross section of $22.3\,\mathrm{nm}^2$.

Correction of the polarizability for particles on a dielectric surface

As the nanoparticles in our experiments lie on the surface of a dielectric mirror, also the effect of a surface close to the particle has to be considered. For metal nanospheres far away from plasmon resonance and for an incident light field with a polarization parallel to the surface, like in the case discussed in this paper, Wind et al. [7] give the following solution:

$$\alpha = \epsilon_0 \frac{\epsilon_1(\epsilon_3 - \epsilon_1)V_p}{\epsilon_1 + L_s(\epsilon_3 - \epsilon_1)} \tag{8}$$

with the depolarization factor

$$L_s = \frac{1}{3}\left(1 - \frac{1}{8}\frac{\epsilon_2 - \epsilon_1}{\epsilon_2 + \epsilon_1}\right). \tag{9}$$

Scattering and Absorption

When light interacts with an object it either gets absorbed or scattered. For nanoparticles smaller than a wavelength of light, this is described by Rayleigh scattering. The corresponding cross sections are given by:

$$C_{abs} = \frac{2\pi\sqrt{\epsilon_1}}{\lambda\epsilon_0}\Im(\alpha) \tag{10}$$

and

$$C_{sca} = \left(\frac{2\pi}{\lambda}\right)^4 \frac{|\alpha|^2}{6\pi\epsilon_0^2}. \tag{11}$$

As both effects attenuate the probe beam, the measured quantity in all our experiments is the extinction of a nanoparticle:

$$C_{ext} = C_{abs} + C_{sca}. \tag{12}$$

Size- and g-factor distribution for gold nanospheres

The size of the nanoparticles varies as well as the g-factor, a heuristic value characterizing damping effects for small nanoparticles. In order to compare our measured distribution of extinction cross sections, we take a Gaussian diameter distribution measured by the manufacturer of the particles with central diameter 41.1 nm and a standard deviation of 3.3 nm. In addition, we adopt a gaussian distribution of the g-factor found by Muskens et. al. [6] around 1.4 with a standard deviation of 0.25.

Calculations for nanorods

For all calculations concerning the polarizability of nanorods, we use the calculus described above for ellipsoids. We correct ϵ_3 for small-size effects of the particle. In equation 6, we use the size of the long (short) axis as particle diameter for the calculation of the polarizability along the respective axis. As we are interested in differences, we do not consider surface effects, which we assume to be equal for both axes.

Supplementary References

[1] Uphoff, M., Brekenfeld, M., Rempe, G. & Ritter, S. Frequency splitting of polarization eigenmodes in microscopic fabry-perot cavities. *New J. Phys.* **17**, 013053 (2015).

[2] Moriwaki, S., Sakaida, H., Yuzawa, T. & Mio, N. Measurement of the residual birefringence of interferential mirrors using Fabry-Perot cavity. *Appl. Phys. B* **65**, 347 (1997).

[3] Brandi, F. *et al.* Measurement of the phase anisotropy of very high reflectivity interferential mirrors. *Appl. Phys. B* **65**, 351 (1997).

[4] van de Hulst, H. *Light Scattering by Small Particles*. Dover Books on Physics Series (Dover Publications, 1957).

[5] Johnson, P. B. & Christy, R. W. Optical constants of the noble metals. *Phys. Rev. B* **6**, 4370–4379 (1972).

[6] Muskens, O. L., Billaud, P., Broyer, M., Del Fatti, N. & Vallée, F. Optical extinction spectrum of a single metal nanoparticle: Quantitative characterization of a particle and of its local environment. *Phys. Rev. B* **78**, 205410 (2008).

[7] Wind, M. M., Vlieger, J. & Bedeaux, D. The polarizability of a truncated sphere on a substrate i. *Physica A: Statistical Mechanics and its Applications* **141**, 33 – 57 (1987).
